# One day of environment‐induced heat stress causes injury to the murine kidney

**DOI:** 10.1113/EP092975

**Published:** 2025-10-02

**Authors:** Melissa Roths, Alyona Michael, Zachary J. Schlader, Joshua T. Selsby

**Affiliations:** ^1^ Department of Animal Science Iowa State University Ames Iowa USA; ^2^ Department of Pathobiology Auburn University Auburn Alabama USA; ^3^ Department of Kinesiology Indiana University School of Public Health–Bloomington Bloomington Indiana USA

**Keywords:** climate change, glomerulus, heat stroke, hyperthermia, tubule

## Abstract

Environment‐induced heat stress (EIHS) results from sustained body temperature elevation owing to prolonged exposure to heat and humidity. We hypothesized that EIHS would cause kidney injury and cellular dysfunction. To test this hypothesis, female C57 mice were exposed to EIHS (*n* = 14; 37.6°C, 42.0% relative humidity) or thermoneutral (TN) conditions (*n* = 12; 31.2°C, 35.0% relative humidity) for 24 h. EIHS increased rectal temperature by 2.1°C (*p *< 0.001), decreased body mass by 10% (*p *= 0.036), decreased absolute kidney mass by 10% (*p *= 0.026), increased renal water content by 19% (*p *< 0.007) and increased blood urea nitrogen by 102% (*p *< 0.001) compared with TN. Histological analysis revealed that EIHS caused proximal tubule vacuolation (*p* = 0.001) and alterations in glomerular structure, supported by markers of lipid accumulation, tubular injury and inflammatory signalling. EIHS increased relative protein abundance of heat shock proteins 70 (48%, *p *= 0.002), 90 (37%, *p *= 0.001) and 60 (36%, *p *= 0.026), in addition to heat shock factor 1 (2‐fold, *p *= 0.020) compared with TN. In addition, there was biochemical evidence of mitochondrial remodelling, increased autophagic flux and robust activation of endoplasmic reticulum stress in kidneys from EIHS mice compared with TN mice. Collectively, these data suggest that 24 h of EIHS is sufficient to cause histological injury and cellular dysfunction, which might undermine renal health and function.

## Abstract (highlights)

- **What is the central question of this study?**
  Does environment‐induced heat stress impair kidney health?
- **What is the main finding and its importance?**
  One day of environment‐induced heat stress is sufficient to alter the architecture of the renal corpuscle and cause cellular injury, which might be reflective of renal thermic injury.

## 1 INTRODUCTION

Prolonged exposure to inescapable heat and humidity can lead to sub‐heat stroke thermic injury, whereby the environmental conditions limit effective heat transfer from an organism to the environment. Current climate conditions, sometimes compounded by the urban heat island effect (Mohammad Harmay & Choi, 2023), pose a threat to human health that is likely to expand given the anticipated increased frequency, severity and duration of heat waves (Stocker et al., 2013). These environmental conditions are a particular health concern for outdoor workers, those with some underlying health conditions, and adults aged ≥35 years (Hansson et al., 2022; Linares & Díaz, 2008; Michelozzi et al., 2005; Moyce et al., 2017; Schlader et al., 2017, 2019; Tawatsupa et al., 2012; Venugopal et al., 2015; Wesseling et al., 2016, 2020). For example, 82% of outdoor workers in India experienced thermic injury in the summer, and remarkably, 42% of workers experienced heat‐related injury in colder seasons (Venugopal et al., 2015). Although heat waves increase the frequency of heat stroke, far more people experience sub‐heat stroke thermic injuries, which we collectively term as being caused by environment‐induced heat stress (EIHS) (Baumgard & Rhoads, 2013), which is also termed heat injury in the literature (Oki et al., 2022). EIHS is responsible for >70% of heat‐related hospitalizations and emergencies (Abdelmoety et al., 2018; Harduar Morano & Watkins, 2017).

Heat stroke has been well documented to cause sustained structural kidney damage (Chalise et al., 2023; Sithinamsuwan et al., 2009; Thongprayoon et al., 2020) and acute kidney injury (AKI) and can lead to chronic kidney disease (CKD) (Donham et al., 2020; Tseng et al., 2020). Although less severe, EIHS has similar renal consequences. For example, 15% of people who work in the heat experience AKI (Flouris et al., 2018), and those who develop AKI and/or CKD are at greater risk of heat‐related hospitalization and/or death (Bobb et al., 2014; Dematte et al., 1998; Gao et al., 2022; Kim et al., 2018; Wu et al., 2021). Notably, these injuries appear to persist, because victims of EIHS are more likely to experience CKD ≤14 years following EIHS (Wang et al., 2019). Despite this general understanding, the consequences of EIHS on kidney health are largely limited to associative data in studies using primarily observational designs.

In our previous work using a porcine EIHS model, we discovered increased blood urea nitrogen (BUN) and serum creatinine, both of which are suggestive of renal injury (Opgenorth et al., 2021; Roths et al., 2023c; Rudolph et al., 2024b). Human models consistently indicate that EIHS increases the risk of developing AKI, but these studies are limited by shorter durations in comparison to those encountered during a naturally occurring EIHS event that could last for days (Chapman et al., 2020; Hess et al., 2023; Houck et al., 2022; McKenna et al., 2024). Furthermore, rodent hyperthermia models generally rely on short durations (<5 h) and excessively hot environmental conditions (>40°C), which more readily replicate heat stroke‐provoking conditions (Chen et al., 2020; Sato et al., 2019); however, this might not recapitulate the consequences of EIHS. Indeed, EIHS can result from unrelenting exposure to hot environmental conditions, often accompanied by hot overnight temperatures, which limit the recovery of body temperatures during the nighttime, and is associated with a heightened likelihood of heat‐related morbidity and mortality (He et al., 2022). Given the current and increasing frequency of persistent hot environmental conditions and the lack of knowledge regarding the impact of EIHS on kidney health, the purpose of this investigation was to determine the extent to which long‐term environment‐induced hyperthermia impairs kidney health. We hypothesized that 24 h of EIHS would cause histopathological injury and cellular dysfunction in the kidneys.

## 2 MATERIALS AND METHODS

### 2.1 Ethical approval, animal treatment and experimental design

The Institutional Animal Care and Use Committee at Iowa State University approved all procedures (IACUC‐20‐134). A detailed description of methods has been published previously (Roths et al., 2024). The investigators understand the ethical principles under which *Experimental Physiology* operates, and our work complies with this animal ethics checklist. All animals were sourced from Jackson Laboratories. Briefly, 26 C57BL6 female mice (4 weeks of age) were housed in groups of three in thermoneutral (TN) conditions and had ad libitum access to food (Prolab RMH 1000, LabDiet) and water. Mice were held in cages with ad libitum access to food and water with minimal disturbance to facilitate acclimation to their new environment for 1 week. After this 1 week period, mice were sedated with isoflurane (5% induction), then quickly implanted with a subcutaneous Unified Information Devices (UID) temperature programmable microchip (UCT‐2112) between the shoulder blades with a needle, allowing measurement of temperature, as previously described (Roths et al., 2024). Animals were monitored for the need of postoperative care or analgesics, but animals did not require additional intervention. At 7 weeks of age, mice were individually housed and assigned randomly to either TN conditions (*n* = 12, 31.2°C ± 1.0°C, 35% ± 1% relative humidity) or EIHS conditions (*n* = 14; 37.6 ± 0.0°C, 42% ± 1% relative humidity) for 24 h. To induce EIHS, conscious, unrestrained mice were held in an environmental chamber (Avantor #76205‐438) for 24 h in two randomly assigned cohorts starting at 11.00 or 12.00 h. All mice had free access to food and water throughout the duration of the environmental challenge. Rectal temperature (RT) was measured every 12 h, and subcutaneous (Sq) temperature was monitored every 4 h. To allow calculation of autophagic flux, 48 and 24 h before the end of the experiment, eight randomly selected mice (*n* = 4 per group) were given an intraperitoneal injection of 0.4 mg/kg colchicine. After the environmental treatment, all mice were killed via an intraperitoneal injection of 0.25 mL phenobarbitone, then the right and left kidneys were removed and weighed. The time interval between removal from environmental conditions and injection with phenobarbitone was ∼5 min. The left kidney was frozen in liquid nitrogen for subsequent biochemical analyses and the right kidney was fixed in 4% paraformaldehyde for histological evaluation.

### 2.2 Tissue water content

Total water content in the kidney was determined using a previously described approach (Roths et al., 2023b, 2024). Briefly, ∼10 mg of the kidney was lyophilized over 48 h. Tissue weight was recorded every 24 h until a consistent measure was found, which occurred at 24 and 48 h. The percentage of water was determined as the percentage difference between the wet weight and the dry weight.

### 2.3 Protein isolation and western blot

Whole homogenate protein was isolated as previously described (Roths et al., 2023a, 2023c, 2024). Briefly, 20 mg of powdered kidney was homogenized in a 1:10 weight‐to‐volume ratio in protein extraction buffer (10 mM sodium phosphate buffer, pH 7.0, 2% SDS, 1% Halt protease and phosphates inhibitor cocktail) and centrifuged (10 621*g* for 15 min at 4°C). After centrifugation, the supernatant was collected, and total protein concentrations were determined using the Pierce BCA Protein Assay Kit (#23227 ThermoFischer Scientific, USA) according to the manufacturer's instructions. Samples were loaded (14 µg protein) onto precast 4%–20% polyacrylamide gels, separated (45 min at 180 V; Bio‐Rad, Hercules, CA, USA) and transferred onto nitrocellulose membranes (100 V for 1 h at 4°C). Equal loading was confirmed on all membranes by quantifying Ponceau S stain (PonS) using whole‐lane densiometry with an Azure Biosystems c600 imaging system. Stain was removed, and membranes were then blocked in 5% dehydrated non‐fat milk in tris‐buffered saline with 0.1% Tween‐20 (TTBS) and probed with primary antibodies (Table 1) overnight at 4°C. Membranes were washed three times for 10 min in TTBS, incubated for 1 h with their respective secondary antibody at room temperature, and washed three times, each for 10 min, in TTBS. Membranes were rocked in ECL for 5 min, and proteins were detected using an Azure Biosystems c600 imaging system. Bands were quantified using automated band detection in the AzureSpot Software (Azure, Dublin, CA, USA).

**TABLE 1:** Antibodies and dilutions used for western blot analysis.

Antibody	Company/product number	Primary dilution	Secondary dilution
Adenosine monophosphate activated kinase (AMPKα)	Cell Signaling Technology (CST), #5882	1:1000	1:1000
Phospho‐AMPKα (Thr172)	CST, #2523	1:1000	1:1000
Activator protein 1 (AP‐1)	Millipore Sigma, A5968	1:1000	1:1000
ATF4 (E4Q4E)	CST, #97038 mouse	1:500	1:1000
ATG7 (D12B11)	CST, #8558	1:1000	1:1000
ATG12/5 (D88H11)	CST, #4180	1:1000	1:1000
ATG16L1 (D6D5)	CST, #8089	1:1000	1:1000
Beclin‐1 (C50B12)	CST, #3495	1:1000	1:1000
BiP (C50B12)	CST, #3177	1:1000	1:1000
BCL2/Adenovirus E1B 19 kDa Protein‐interacting protein 3‐like (BNIP3L/Nix)	CST, #12396	1:500	1:1000
CHOP (D46F1)	CST, #5554	1:500	1:1000
Dynamic‐related protein 1 (DRP1) (D6C7)	CST, #8570	1:1000	1:1000
Phospho‐dynamin‐related protein 1 (DRP1) (D6C7)	CST, #8570	1:1000	1:1000
FIS1	Proteintech, #10956	1:1000	1:1000
Heat shock protein 60 (HSP60) (D6F1) XP(R)	CST, #12165	1:1000	1:1000
Heat shock protein 70 (HSP70)	Novus, NB110‐96427 mouse	1:1000 (5% milk)	1:1000
Heat shock protein 90 (HSP90)	CST, #4877	1:1000	1:1000
Heat shock factor 1 (HSF1)	CST, #12972	1:1000	1:1000
Interleukin (IL)‐6	Abcam, ab6672	1:1000	1:1000
Interleukin (IL)‐10	Santa Cruz Technologies, SC‐8438 mouse	1:1000	1:1000
Inositol‐requiring enzyme type 1 (IRE1α) (14C10)	CST, #3294	1:1000	1:1000
Phospho‐ IRE1α (S724)	Abcam, ab48187	1:1000	1:1000
c‐Jun N‐terminal kinases (JNK)	CST, #9252	1:1000	1:1000
Phosphorylated c‐Jun N‐terminal kinases (p‐JNK) (Thr183/Tyr185) (81E11)	CST, #4668	1:500	1:1000
Liver‐type fatty acid‐binding protein (L‐FABP)	SC‐ 271591 mouse	1:1000	1:1000
Monocyte chemoattractant protein‐1 (MCP‐1)	SC‐ 52701 mouse	1:1000	1:1000
Mitofusion‐1	Proteintech, #66776 mouse	1:1000	1:1000
Mitofusion‐2	SC‐515647 mouse	1:500	1:1000
Microtuble‐associate protein light chain (LC3 A/B)	CST, #12741	1:500 (2.5% milk)	1:1000
Nuclear factor kappa B (NFκB p65) (D14E12) XP (R)	CST,# 8242	1:1000	1:1000
Phosphorylated nuclear factor kappa B (p‐NFκB) (Ser536) (93H1)	CST, #3033	1:500	1:1000
Neutrophil gelatinase‐associated lipocalin (NGAL)	SC‐515876 mouse	1:1000	1:1000
Optic atrophy 1 (OPA1)	Abcam, ab157457	1:1000	1:1000
Sequestosome‐1 SQSTM1/p62	Abcam, ab109012	1:500	1:1000
Parkin	SC‐32282 mouse	1:1000	1:1000
Perilipin (PLIN) 1	Novus, NB100‐60554 goat	1:1000	1:2000
Perilipin (PLIN) 2	Novus, NB110‐40877	1:1000	1:1000
Peroxisome proliferator‐activated receptor alpha (PPARα)	Proteintech, 66826‐1 mouse	1:1000	1:1000
Peroxisome proliferator‐activated receptor gamma (PPARγ)	Proteintech, 16643‐1	1:1000	1:1000
PI3 kinase class III (PI3K)	CST, #3358	1:1000	1:1000
Protein kinase‐like endoplasmic reticulum kinase (PERK)	Proteintech, #24390	1:1000	1:1000
Phospho‐PERK (Ser719)	Proteintech, #29546	1:1000	1:1000
PTEN‐induced kinase 1 (PINK1)	CST, #6946	1:1000	1:1000
Toll‐like receptor 4 (TLR4)	SC‐ 293072 mouse	1:1000	1:1000
Tumor necrosis factor alpha (TNFα)	Invitrogen, # PA5‐77317	1:500	1:1000
ULK1 (D8H5)	CST, #8054	1:1000	1:1000
Phospho‐ULK1 (Ser555) (D1H4)	CST, #5869	1:500	1:1000
XBP‐1S (E9V3E)	CST, #40435	1:500	1:1000

### 2.4 Histology

The kidney was fixed in 4% paraformaldehyde for 24 h, then stored in 70% ethanol. Fixed tissues were paraffin embedded. Sections 4 µg thick were cut and mounted on a slide, then stained with Haematoxylin and Eosin by the Iowa State University Veterinary Diagnostic Laboratory. Sections were imaged and subjectively scored for injury by a blinded, board‐certified, veterinary anatomical pathologist. For the subjective evaluation, the entirety of the kidney was viewed. Vacuolation of the proximal convoluted tubule was assigned a score as follows: 1 = absent, 2 = mild, focal, 3 = moderate, or 4 = marked.

In addition, we objectively quantified several parameters related to renal corpuscle structure in 10 randomly selected glomeruli per kidney per animal using Aperio ImageScope. Initially, we measured the area of each corpuscle fully contained within each image, then, separately, measured the area of the corresponding glomerulus. The area of Bowman's space was calculated as the corpuscle area minus the glomerular area. Finally, the glomerular area and Bowman's space area were expressed as a percentage of the total corpuscle area. The number of nuclei per image was counted on two ×20 magnification images per animal using density slicing in OpenLab.

### 2.5 Blood urea nitrogen

Immediately following euthanasia, the heart was removed, blood collected from the thoracic cavity, and allowed to clot at room temperature for 30 min. After 30 min, blood was centrifuged at 2000*g* for 10 min at 4°C, and serum was collected and stored at −80°C until use. Stored serum extracts (20 µL) were diluted in 80 µL deionized water, for a total of 100 µL. Owing to sample limitations, seven TN and four EIHS samples were used for this test. The BUN was read on a Vetscan VS2 Chemistry analyser (rotor 1002324; Zoetis).

### 2.6 Statistics

Western blot and histological data were compared using Student's unpaired two‐tailed *t*‐test with GraphPad Prism statistical software. Data are presented as means ± SD. Colchicine‐treated and non‐colchicine‐treated groups were analysed using the proc MIXED procedure in SAS v.9.4 (SAS Institute Inc., Cary, NC, USA). The model consisted of main effects of environment and colchicine treatment, and data are presented as means ± SD. Data points >2SD away from the mean were excluded regardless of group or direction, and such instances are noted in the Results section and figure legends. Significance was established as *p *< 0.05.

## 3 RESULTS

**FIGURE 1:**
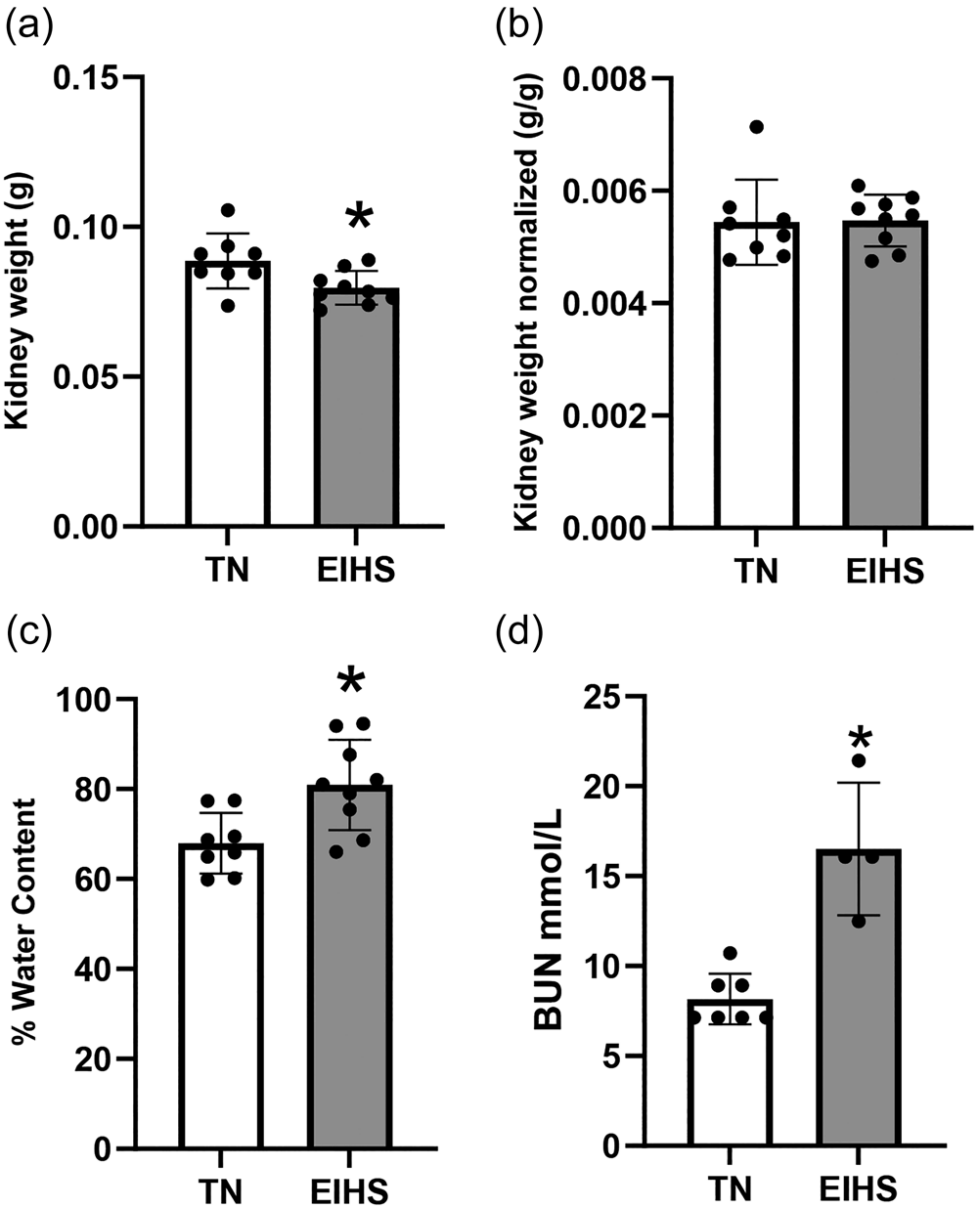
EIHS caused kidney architecture to change and increased BUN. (a) EIHS caused kidney weight to decrease in comparison to TN (*p *= 0.026). (b) However, kidney weight normalized to body weight was similar between groups (*p *= 0.930). (c) The water content of kidneys recovered from EIHS mice was greater than from TN mice (*p *< 0.007). (d) BUN was increased in EIHS mice compared with TN (*p *< 0.001). Results are expressed as means ± SD. *p < 0.05. (a–c) TN = 8 per group and EIHS = 9 per group. (d) Owing to limited sample amount, TN = 7 per group and EIHS = 4 per group. Abbreviations: BUN, blood urea nitrogen; EIHS, environment‐induced heat stress; TN, thermoneutral.

**FIGURE 2:**
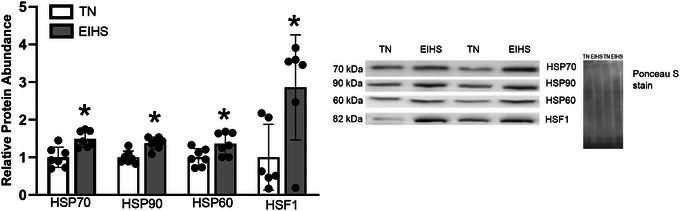
The effect of environment‐induced heat stress on heat shock proteins in the kidney. HSPs 70 (*p *= 0.002), 90 (*p *< 0.001) and 60 (*p *= 0.026) and HSF1 (*p *= 0.020) were increased by EIHS compared with thermoneutral (TN) conditions. Results are expressed as means ± SD, *p < 0.05. *n* = 7 per group; one outlier was removed from TN and EIHS HSF1. Abbreviations: EIHS, environment‐induced heat stress; HSF1, heat shock factor‐1; HSP, heat shock protein; TN, thermoneutral.

A detailed report of the physiological response to this heat intervention has been reported previously (Roths et al., 2024). Briefly, heat exposure elevated RT by ∼2.1°C (SD ±0.2°C) and Sq temperature by ∼1.8°C (SD ±0.2°C) and decreased body mass by 10% (*p *= 0.036). Environment‐induced heat stress decreased absolute kidney mass by ∼10% (*p *= 0.026) compared with TN; however, normalized kidney mass was similar between groups (*p *= 0.930; Figure 1). We next measured kidney water content and discovered that water content was increased by 19% (*p *< 0.007) in EIHS compared with TN (Figure 1). BUN was increased by 102% (*p *< 0.001) in EIHS compared with TN (Figure 1). Our environmental treatment increased relative protein abundance of heat shock proteins (HSPs) 70 (48%, *p *= 0.002), 90 (37%, *p *< 0.001) and 60 (36%, *p *= 0.026), in addition to heat shock factor 1 (HSF1) (2‐fold, *p *= 0.020; one outlier removed from TN and EIHS) compared with TN (Figure 2).

**FIGURE 3:**
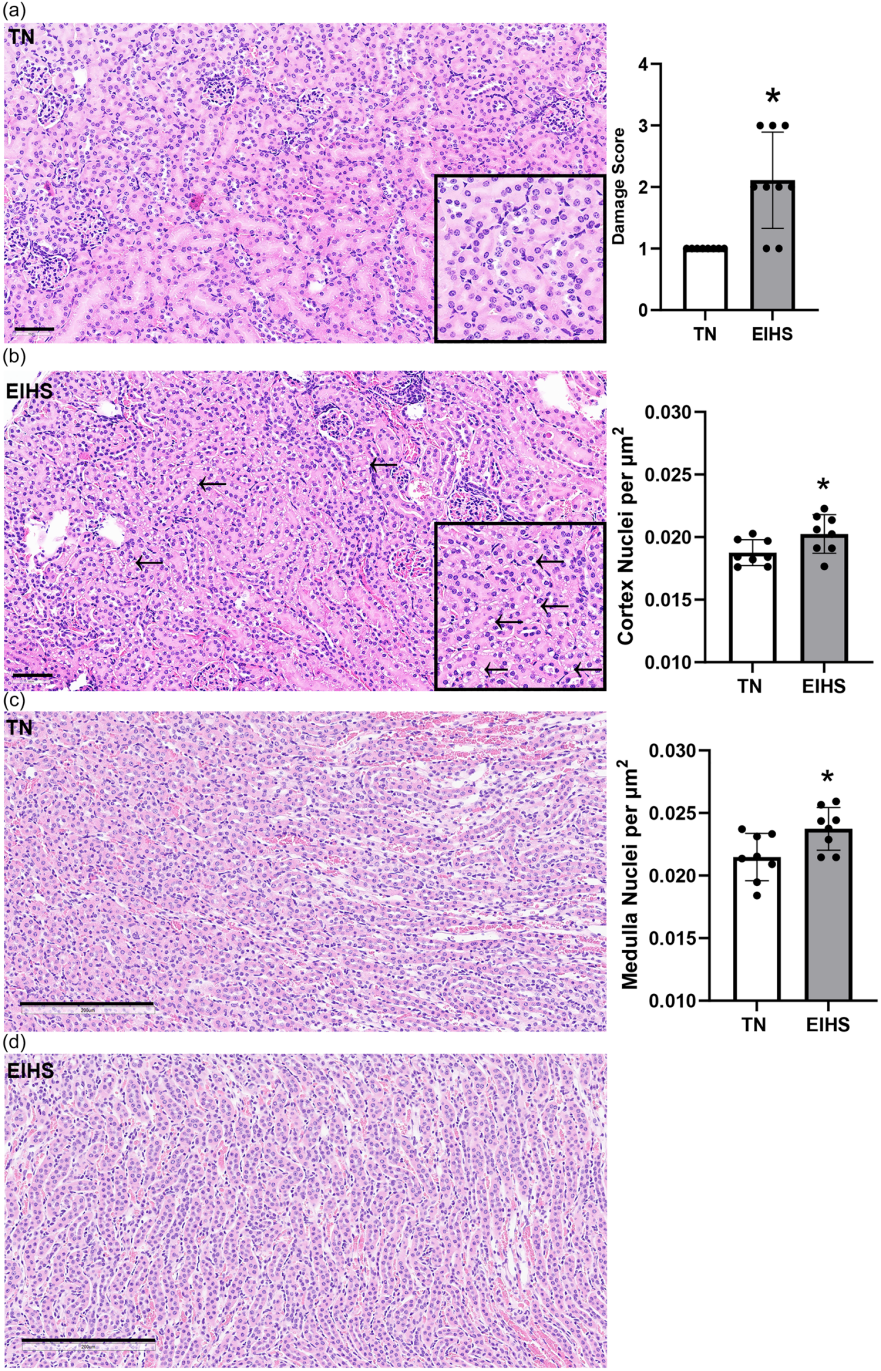
Histological evaluation of the kidney after EIHS. Representative ×40 images of the kidney in TN (a) and EIHS (b) conditions. A trained, blinded pathologist performed a subjective evaluation of changes in the kidney. For the subjective evaluation, the entirety of the kidney was viewed. Lesions were assigned a score, as follows: 1 = absent, 2 = mild, 3 = moderate,= or 4 = marked, widely distributed. Histological inspection suggested increased vacuolation following 24 h of EIHS compared with TN conditions (*p *< 0.001). In addition, the number of nuclei was quantified. The number of nuclei was increased in the cortex (*p = *0.039) (b) and medulla (*p *= 0.025) (d) following EIHS compared with TN cortex (a) and TN medulla (c). Representative images of medulla under TN (c) or EIHS (d) conditions are ×20 images. Scale bars: 60 µm in (a) and (b); 200 µm for (c) and (d). Black arrows indicate vacuolation. Inset images are digitally magnified at 250%. *p < 0.05. Abbreviations: EIHS, environment‐induced heat stress; TN, thermoneutral.

**FIGURE 4:**
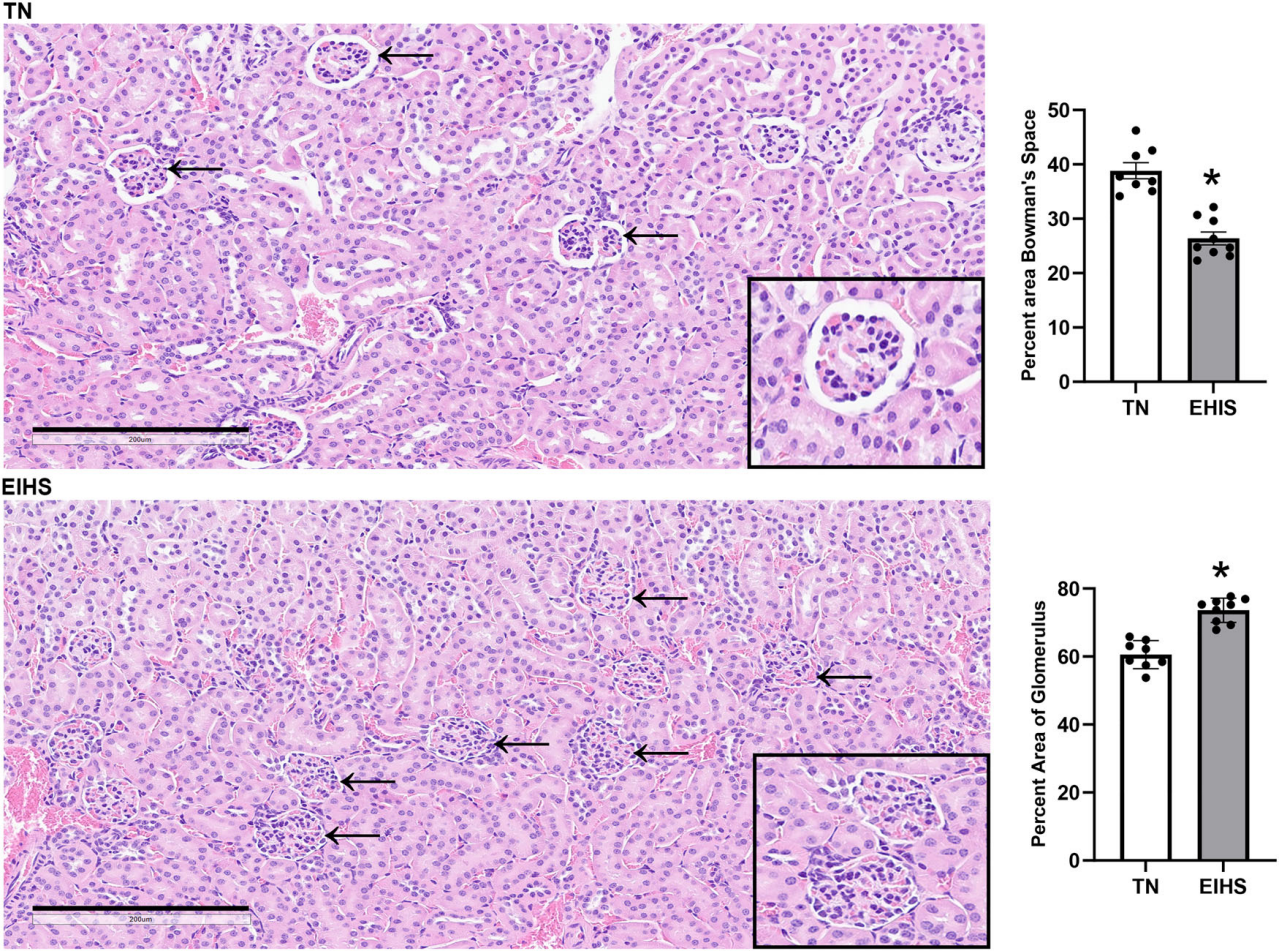
Kidney structure changed after 24 h of EIHS. Representative ×20 images of the kidney with and without EIHS. Scale bars: 200 µm. Black arrows indicate renal corpuscles. Inset images are digitally magnified at 250%. Renal corpuscle structure was quantified objectively in 10 randomly selected renal corpuscles per animal (×20 magnification). The area of Bowman's space was calculated as the corpuscle area minus the glomerular area. The glomerular area and Bowman's space area were expressed as a percentage of the total corpuscle area. Relative glomerular area was significantly increased (*p *< 0.001) and Bowman's space significantly decreased (*p* < 0.001) by EIHS. *p < 0.05. Abbreviations: EIHS, environment‐induced heat stress; TN, thermoneutral.

To determine the extent to which EIHS might damage the kidney, we next performed a histological inspection of the kidney. We discovered that kidneys from EIHS animals had increased vacuolation in the proximal convoluted tubules (*p *< 0.001) compared with kidneys from TN animals (Figure 3). The number of nuclei in the cortex increased by 8% (*p = *0.039) and in the medulla by 10% (*p *= 0.025) in EIHS compared with TN (Figure 3). The absolute areas of the renal corpuscle and glomerulus were similar between groups; however, when considered relative to total corpuscle area, glomerular area was increased by 21% (*p *< 0.001) and Bowman's space was decreased by 32% (*p* < 0.001) in EIHS compared with TN (Figure 4).

**FIGURE 5:**
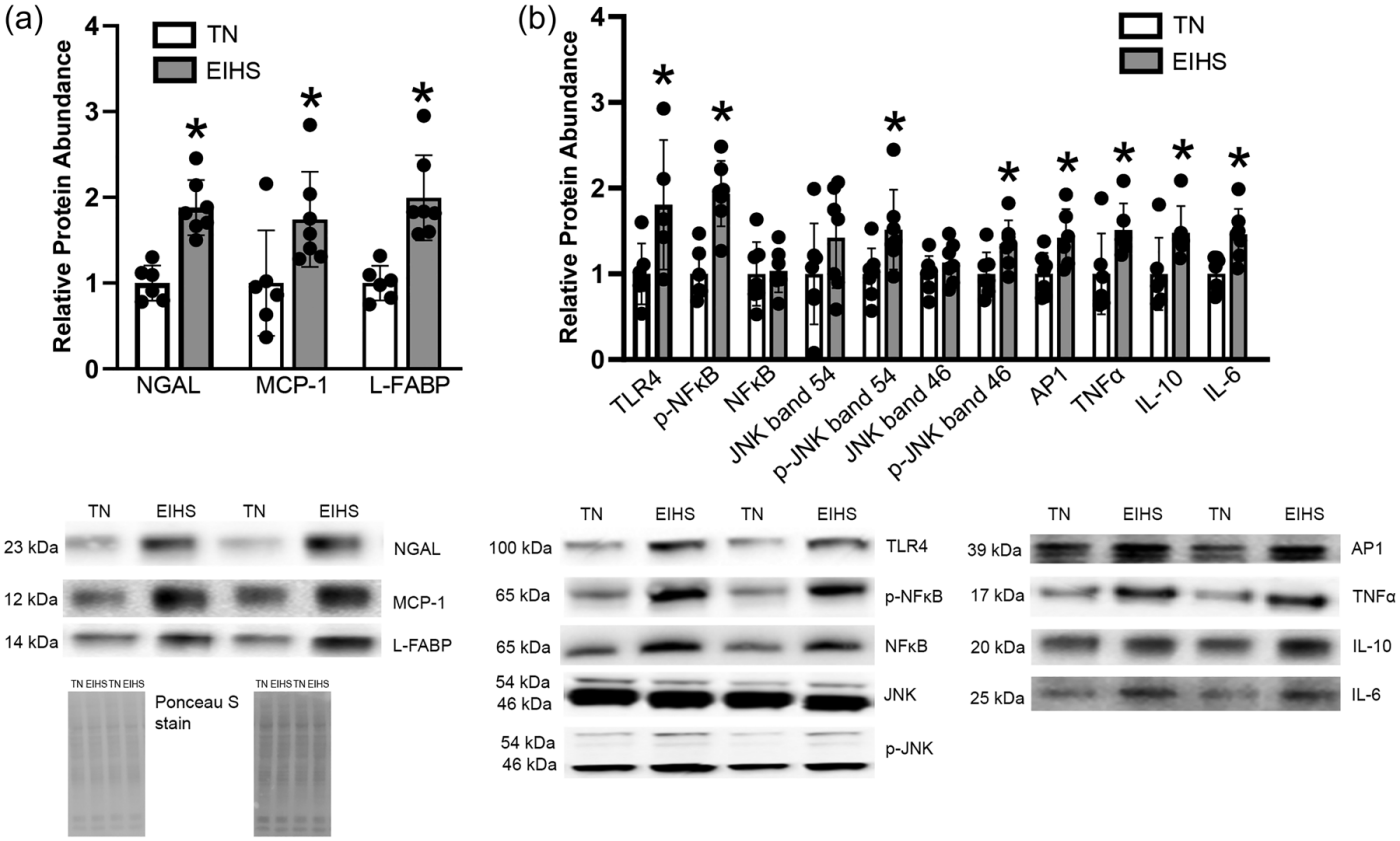
Effects of EIHS on kidney injury markers and inflammatory markers. (a) Relative protein abundances of kidney injury markers NGAL (*p *< 0.001), MCP‐1 (*p *= 0.042) and L‐FABP (*p *< 0.001) were increased by EIHS compared with TN conditions. (b) EIHS increased relative protein abundance of TLR4 (*p *= 0.043), p‐NFKB (*p *< 0.001), p‐JNK (*p *= 0.029), AP1 (*p *= 0.023), TNFα (*p *= 0.050), IL‐10 (*p *= 0.050) and IL‐6 (*p *= 0.005) compared with TN; however, total NFKB (*p *= 0.838) and total JNK (54 kDa, *p *= 0.204 and 46 kDa, *p *= 0.287) were similar between groups. Results are expressed as means ± SD. *p < 0.05. (a) TN = 7 per group and EIHS = 7 per group, with one outlier removed from TN NGAL, MCP‐1 and L‐FABP. (b) TLR4, TN = 6 per group and EIHS = 5 per group; pNFKB, TN = 7 per group and EIHS = 7 per group, with one outlier removed from TN; NFKB, JNK, p‐JNK and IL‐6, TN = 7 per group and EIHS = 7 per group; AP‐1, TN = 7 per group and EIHS = 7 per group, with one outlier removed from EIHS; and TNFα and IL‐10, TN = 6 per group and EIHS = 6 per group. Abbreviations: EIHS, environment‐induced heat stress; TN, thermoneutral.

**FIGURE 6:**
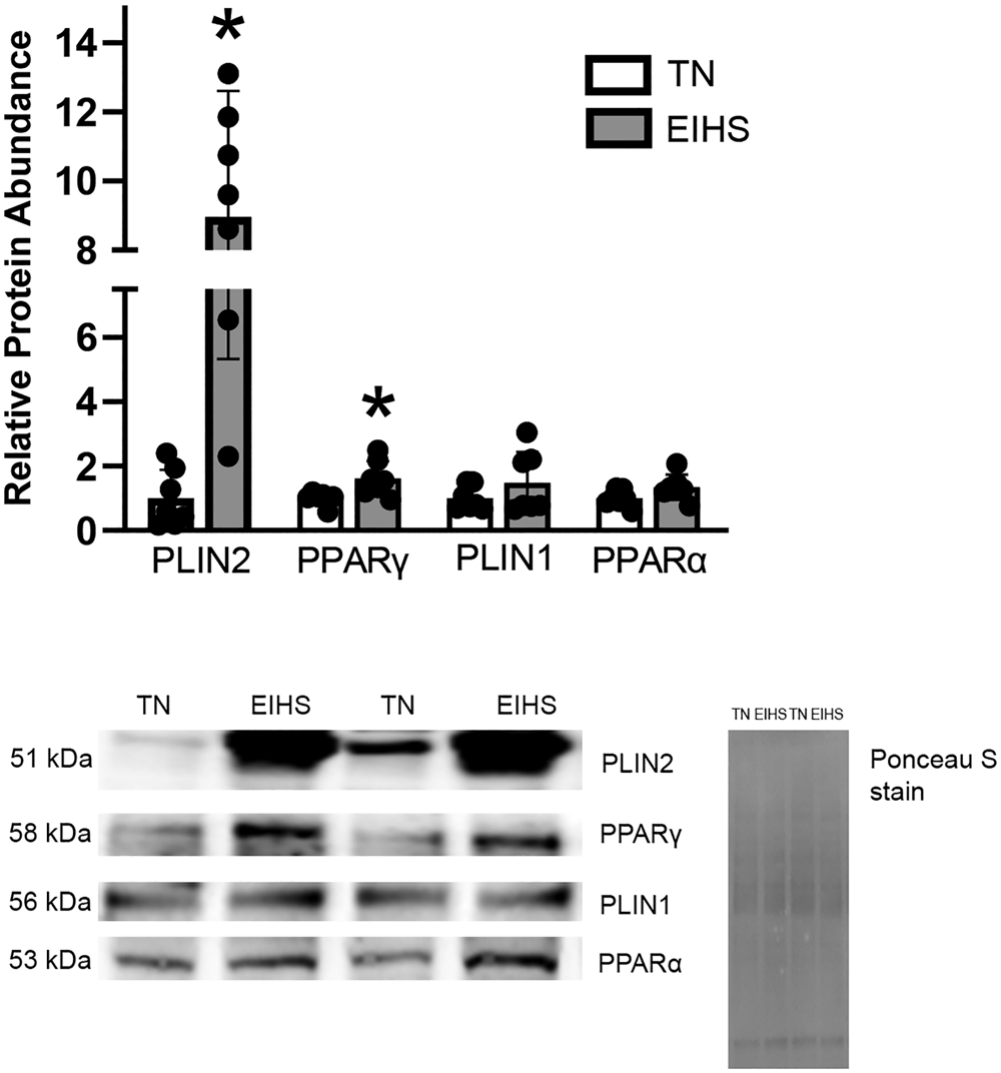
Effects of EIHS on markers of lipid droplets in the kidney. Relative protein abundance of PLIN2 (*p *< 0.001), PLIN3 (*p *< 0.001) and PPARγ (*p *= 0.026) increased following EIHS compared with TN conditions; however, PLIN5 (*p *= 0.195), PLIN1 (*p *= 0.247) and PPARα (*p *= 0.076) were similar between groups. Results are expressed as means ± SD. *p < 0.05. TN = 7 per group and EIHS = 7 per group. One outlier was removed from EIHS PLIN3 and one from TN PPARγ. Abbreviations: EIHS, environment‐induced heat stress; TN, thermoneutral.

Given the apparent histological changes, we next considered biomarkers of kidney tubular injury. EIHS increased relative protein abundance of neutrophil gelatinase‐associated lipocalin (NGAL; 88%, *p *< 0.001; one outlier removed from TN), monocyte chemoattractant protein‐1 (MCP‐1; 74%, *p *= 0.042; one outlier removed from TN) and liver‐type fatty acid‐binding protein (L‐FABP; 99%, *p *< 0.001; one outlier removed from TN) compared with TN (Figure 5a), which are suggestive of tubular injury (Zhang & Parikh, 2019). We also considered inflammatory signalling markers and discovered that the relative protein abundance of Toll‐like receptor 4 (TLR4) and phosphorylated (p)‐nuclear factor kappa B (p‐NFκB) (Ser536) (93H1) were increased by 44% (*p *= 0.043) and 93% (*p *< 0.001; one outlier removed from TN), respectively, by EIHS compared with TN; however, total NFκB was similar between groups (*p *= 0.838; Figure 5b). The pro‐inflammatory transcription factor, activator protein 1 (AP‐1), is phosphorylated by c‐Jun N‐terminal kinases (JNK) (Morgan & Nicolas, 2018). Environment‐induced heat stress increased relative protein abundance of p‐JNK (Thr183/Tyr185) (81E11) (54 kDa; 51%, *p *= 0.029) and p‐JNK (Thr183/Tyr185) (81E11) (46 kDa, 35%, *p *= 0.027) without changes in total JNK (54 kDa, *p *= 0.204 and 46 kDa, *p *= 0.287; Figure 5b). Furthermore, EIHS increased relative protein abundance of AP‐1 (33%, *p *= 0.023; one outlier removed from EIHS), tumor necrosis factor alpha (TNFα; 39%, *p *= 0.050), interleukin (IL)‐10 (36%, *p *= 0.050) and IL‐6 (46%, *p *= 0.005) compared with TN (Figure 5b). Finally, we discovered that EIHS increased relative protein abundance of perilipin (PLIN) 2 (7.9‐fold, *p *< 0.001), PLIN 3 (30%, *p *< 0.001) (one outlier removed from EIHS) and peroxisome proliferator‐activated receptor gamma (PPARγ; 61%, *p *= 0.026; one outlier removed from TN) compared with TN, and that relative protein abundance of peroxisome proliferator‐activated receptor alpha (PPARα; *p *= 0.076) was similar between groups (Figure 6).

**FIGURE 7:**
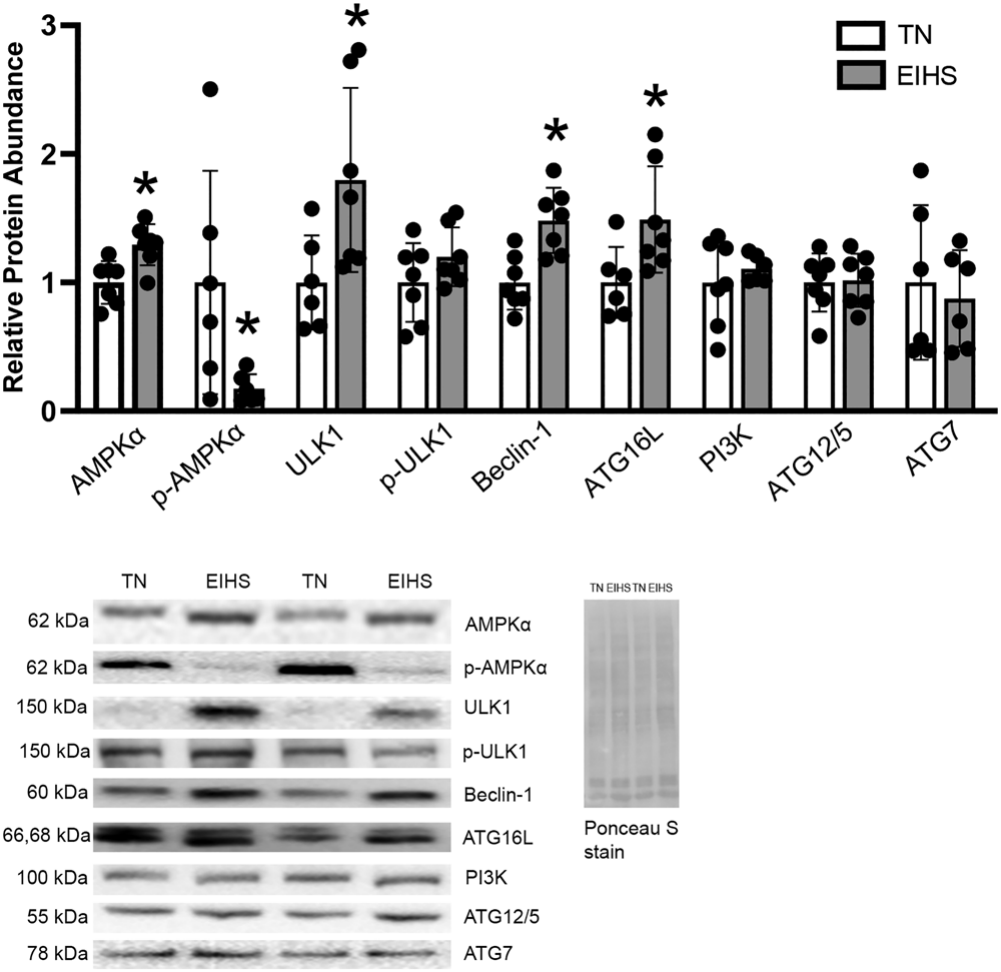
Effects of EIHS on autophagy‐regulatory proteins in the kidney. Relative protein abundances of AMPKα (*p *= 0.005), ULK1 (*p *= 0.032), Beclin‐1 (*p *= 0.002) and ATG16L (*p *= 0.031) were increased by EIHS compared with TN conditions, and p‐AMPKα (*p *= 0.043) was decreased by EIHS compared with TN. Relative protein abundances of p‐ULK1 (*p *= 0.192), PI3K (*p *= 0.475), ATG12/5 (*p *= 0.889) and ATG7 (*p *= 0.674) were similar between groups. Results are expressed as means ± SD. *p < 0.05. TN = 7 per group and EIHS = 7 per group. One outlier was removed from TN ULK1 and ATG16L, and one from EIHS PI3K. p‐AMPKα and ATG7, TN = 6 per group and EIHS = 6 per group. Abbreviations: EIHS, environment‐induced heat stress; TN, thermoneutral.

**FIGURE 8:**
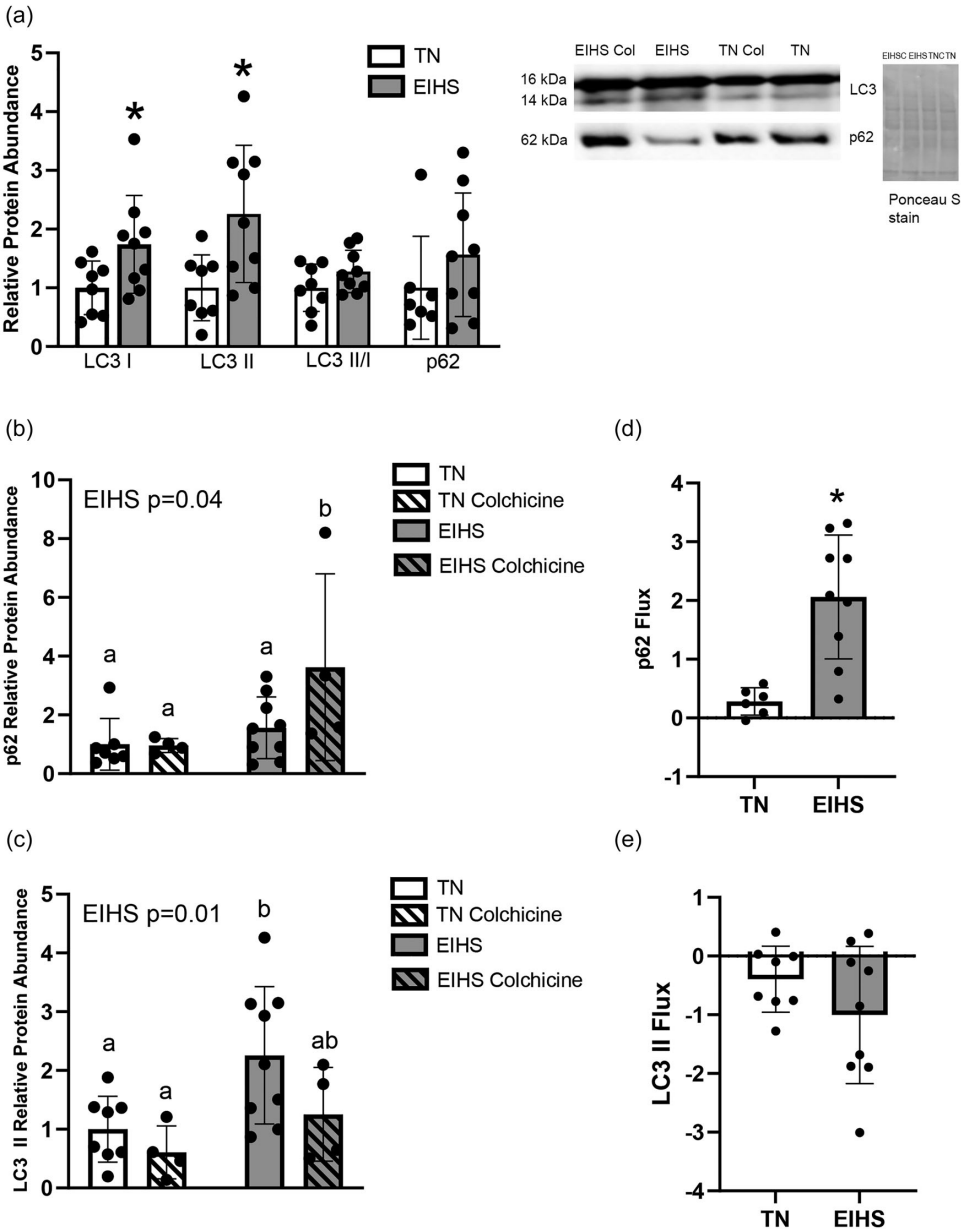
EIHS altered autophagic flux in the kidney. (a) EIHS increased relative protein abundances of LC3 I (*p *= 0.042) and LC3 II (*p *= 0.014) compared with TN conditions, while LC II/I (*p *= 0.151) and p62 (*p *= 0.274) were similar between groups. Results are expressed as means ± SD. *p < 0.05. (b) A subset of mice was treated with colchicine to allow calculation of flux (*n* = 4 per group). There was a main effect of EIHS on relative protein abundance of p62 (*p *< 0.049), and colchicine increased relative protein abundance of p62 in EIHS colchicine‐treated animals compared with EIHS control animals (*p *= 0.031). (c) When considering LC3 A/B II accumulation caused by colchicine, there was a main effect of EIHS (*p *= 0.011), and relative protein abundance of LC3 A/B II was increased in EIHS animals compared with TN control and TN colchicine‐treated animals. (d, e) EIHS increased flux as assessed by p62 (*p *= 0.001; d), but LC3 II was similar between groups (*p *= 0.200; e). Data are shown as means ± SD. TN white striped bars = thermoneutral colchicine‐treated animals; EIHS grey striped bars = environment‐induced heat stress colchicine‐treated animals. *p < 0.05. TN = 8 per group and EIHS = 9 per group. One outlier was removed from TN p62. *n* = 4 per group with colchicine. Abbreviations: EIHS, environment‐induced heat stress; TN, thermoneutral.

To determine the extent to which EHIS affects initiation of autophagy in the kidney, we measured the relative protein abundance of several key regulators of autophagy. EIHS increased relative protein abundance of total adenosine monophosphate‐activated kinase (AMPKα; 29%, *p *= 0.005), but decreased p‐AMPKα (T172) (40H9) (41%, *p *= 0.043; Figure 7). UNC‐51‐like kinase 1 (ULK1) was increased (79%, *p *= 0.032; one outlier removed from TN) by EIHS compared with TN, and p‐ULK1 (Ser555) (D1H4) was similar between groups (*p *= 0.192; Figure 7). Furthermore, we measured the relative protein abundance of markers of autophagosome nucleation and elongation. EIHS increased beclin‐1 (48%, *p *= 0.002) and autophagy‐related gene (ATG) ATG16L (48%, *p *= 0.031; one outlier removed from TN) compared with TN (Figure 7), whereas PI3 kinase class III (PI3K; *p *= 0.475), ATG12/5 (*p *= 0.889) and ATG7 (*p *= 0.674) were similar between groups (Figure 7; one outlier removed from EIHS PI3K). Microtuble‐associated protein light chain (LC3 A/B) I increased by 73% (*p *= 0.042) and LC3 A/B II 1.25‐fold (*p *= 0.014) by EIHS compared with TN; however, LC3 A/B II/I and sequestosome‐1 SQSTM1/p62 (p62) were similar between groups (*p *= 0.151 and *p *= 0.274, respectively; Figure 8a).

In addition, to appreciate the extent to which EIHS altered autophagic flux in the kidney, a subset of mice was treated with colchicine (Carter et al., 2018; Vainshtein et al., 2015). We discovered a main effect of EIHS on relative protein abundance of p62 (*p *< 0.049; one outlier removed from TN) and that colchicine increased relative protein abundance of p62 in EIHS compared with EIHS control animals (2‐fold, *p *= 0.031; Figure 8b). When we considered LC3 A/B II accumulation caused by colchicine, we discovered a main effect of EIHS (*p *= 0.011; Figure 8c). The accumulation of LC3 II and p62 owing to colchicine allows the calculation of flux. EIHS increased flux as assessed by p62 (*p *= 0.001; Figure 8d); however, flux assessed by LC3 II accumulation was similar between groups (*p *= 0.200; Figure 8e).

**FIGURE 9:**
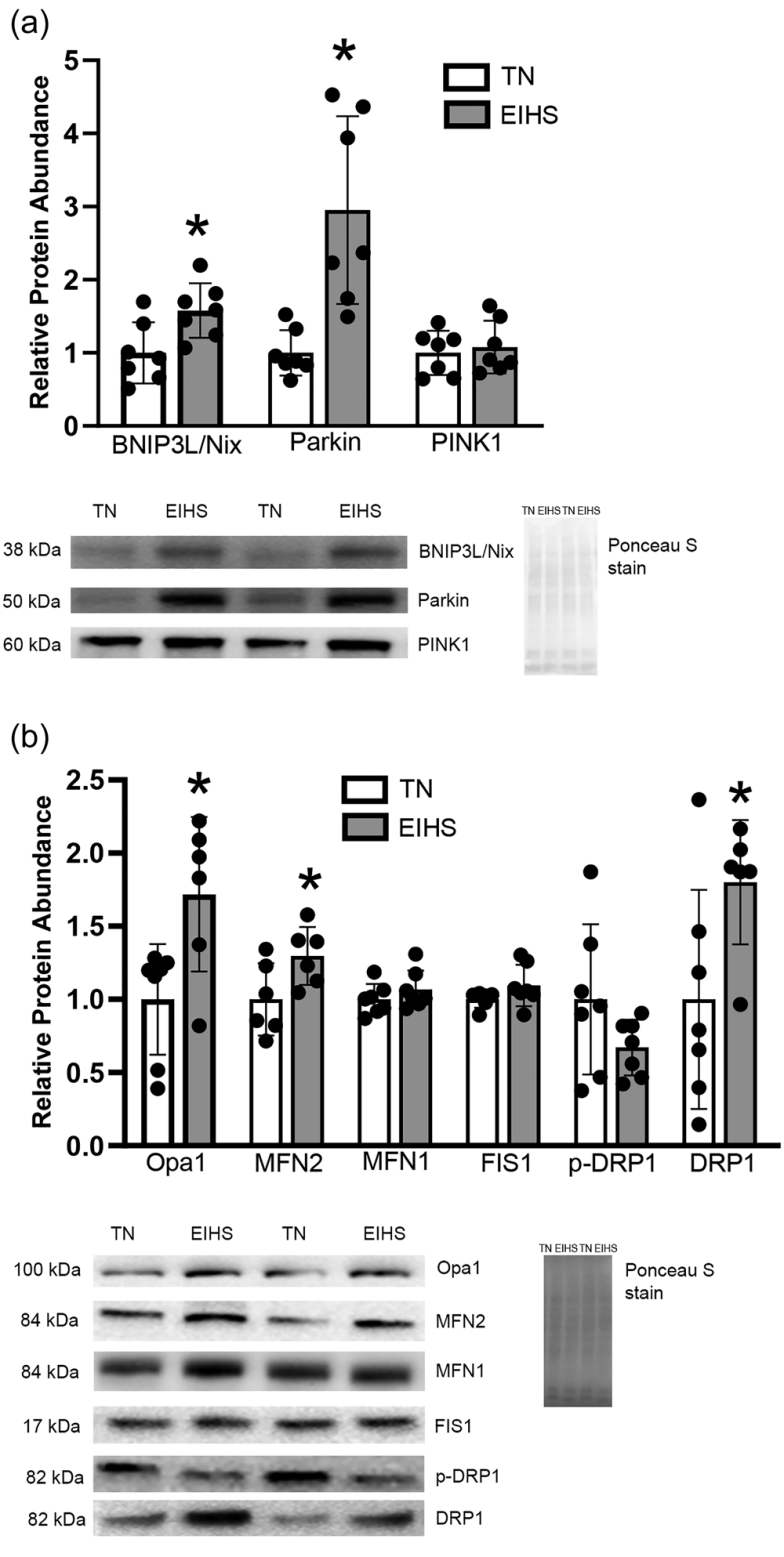
Effects of EIHS on renal mitophagy and regulation of mitochondrial architecture. (a) Relative protein abundances of BNIP3/Nix (*p *= 0.018) and Parkin (*p *= 0.002) increased following EIHS compared with TN conditions, whereas PINK1 was similar between groups (*p *= 0.664). (b) Relative protein abundances of Opa1 (*p *= 0.015), MFN2 (*p *= 0.044) and DRP1 (*p *= 0.041) increased following EIHS compared with TN, and MFN1 (*p *= 0.311), FIS1 (*p *= 0.152) and p‐DRP1 (*p *= 0.138) were similar between groups. Results are expressed as means ± SD. *p < 0.05. (a) TN = 7 per group and EIHS = 7 per group. (b) TN = 7 per group and EIHS = 7 per group. One outlier was removed from EIHS Opa1 and DRP1, and one from TN FIS1. MFN2 TN = 6 per group and EIHS = 6 per group. Abbreviations: EIHS, environment‐induced heat stress; TN, thermoneutral.

To gain insight into how EIHS might impact mitochondrial health, we measured markers of mitophagy, in addition to proteins that participate in mitochondrial fusion and fission. EIHS increased BCL2/Adenovirus E1B 19 kDa protein‐interacting protein 3‐like (BNIP3L/Nix) (57%, *p *= 0.018) and Parkin (195%, *p *= 0.002) compared with TN; however, PTEN‐induced kinase 1 (PINK1) was similar between groups (*p *= 0.664; Figure 9a). Mitophagy‐mediated elimination of damaged mitochondria is linked to mitochondrial fusion and fission processes (Bhatia & Choi, 2019; Onishi et al., 2021). The mitofusion proteins optic atrophy 1 (OPA1) and mitofusin 2 (MFN2) were increased by 71% (*p *= 0.015; one outlier removed from EIHS) and 29% (*p *= 0.044), respectively, by EIHS compared with TN, whereas mitofusion 1 (MFN1) was similar between groups (*p *= 0.311; Figure 9b). The mitofission proteins mitochondrial fission protein 1 (FIS1; one outlier removed from TN) and phospho‐dynamic‐related protein 1 (p‐DRP1) (Ser616) (D9A1) were similar between groups (*p *= 0.152; *p *= 0.138), although EIHS increased total DRP1 (59%, *p *= 0.041; one outlier removed from EIHS) compared with TN (Figure 9b).

**FIGURE 10:**
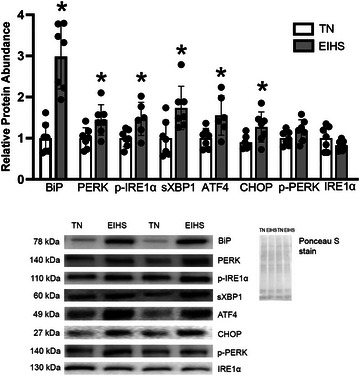
Effects of EIHS on markers of ER stress in the kidney. Relative protein abundances of BiP (*p *< 0.001), Perk (*p *= 0.022), p‐IRE1α (*p *= 0.020), sXBP1 (*p *= 0.014), ATF4 (*p *= 0.021) and CHOP (*p *= 0.044) increased following EIHS compared with TN conditions, and p‐Perk (*p *= 0.137) and IRE1α (*p *= 0.169) were similar between groups. Results are expressed as means ± SD. *p < 0.05. TN = 7 per group and EIHS = 7 per group. One outlier was removed from EIHS ATF4 and p‐IREα, and one from TN CHOP. Abbreviations: EIHS, environment‐induced heat stress; ER, endoplasmic reticulum; TN, thermoneutral.

Accumulation of unfolded proteins can lead to endoplasmic reticulum (ER) stress and activation of the unfolded protein response (UPR) (Rashid et al., 2015; Rutkowski & Hegde, 2010). EIHS increased relative protein abundance of immunoglobulin heavy chain binding protein (BiP; 1.9‐fold, *p *< 0.001), protein kinase‐like endoplasmic reticulum kinase (PERK; 44%, *p *= 0.022) and phosphorylated inositol‐requiring enzyme type 1(p‐IRE1α) (S724) (40%, *p *= 0.020; one outlier removed from EIHS) compared with TN (Figure 10), in addition to the downstream effectors, transcriptional factor spliced X‐box binding protein 1 (sXBP1; 73%, *p *= 0.014), activating transcription factor 4 (ATF4; 40%, *p *= 0.021; one outlier removed from EIHS) and C/EBP homologous protein (CHOP; 41%, *p *= 0.044; one outlier removed from TN; Figure 10). Relative protein abundance of p‐PERK (S719) and total IRE1α was similar between groups (*p *= 0.137 and *p *= 0.169, respectively; Figure 10).

## 4 DISCUSSION

Prolonged exposure to high environmental temperatures is a present and expanding threat to human health. Given the increasing frequency, duration and intensity of extreme heat events, gaining an understanding of the extent to which EIHS impacts kidney health is essential. Although others have reported heat stroke‐mediated AKI and CKD (Donham et al., 2020; Tseng et al., 2020), the effects of thermic injury caused by a single EIHS exposure on the kidneys are largely unknown. Ethical considerations limit the duration of heating during human experimentation, which prevents accurate recapitulation of natural extreme heat events that result in sustained hyperthermia. In our swine model, we have previously reported increased serum creatinine and BUN (Opgenorth et al., 2021; Roths et al., 2023c; Rudolph et al., 2024b), which are suggestive of impaired kidney function and the potential for kidney injury. Given our previous findings, we hypothesized that EIHS would cause histopathological injury and cellular dysfunction in the kidneys.

The environmental intervention used herein increased RT by ∼2°C, which is consistent with temperature elevations that cause sub‐heat stroke thermic injuries (Abdelmoety et al., 2018). EIHS also decreased body mass, which is consistent with our previous work using a porcine EIHS model (Roths et al., 2023c). Of interest, EIHS decreased absolute kidney mass, and this change was consistent in magnitude relative to the observed reductions in body mass. Throughout EIHS, mice had free access to food and water; however, we cannot eliminate dehydration and decreased food intake as underlying factors in changes in body and/or kidney mass. Importantly, like EIHS‐mediated changes to a variety of physiological parameters (i.e., haemodynamics, metabolism, endocrine factors, etc.), their independent and interdependent effects are inseparable consequences of naturally occurring EIHS and are likely to contribute to outcomes reported herein. Previous reports are equivocal regarding EIHS‐mediated changes in kidney mass, with a reduction noted in a porcine model (Cruzen et al., 2015) and an increase reported in a rabbit model (Mutwedu et al., 2021). Complicating the interpretation of these data, however, was our discovery of increased renal water content in heat‐stressed animals, raising the possibility of greater loss of functional kidney mass than anticipated by considering mass alone. Such a loss in functional mass is consistent with increased BUN reported herein and with our observations of a rise in serum creatinine and BUN in our previous work (Opgenorth et al., 2021; Roths et al., 2023c; Rudolph et al., 2024b) and in other animal models (Pearce et al., 2012; Sato et al., 2019; Yunianto et al., 1997). Furthermore, elevated BUN has been documented in humans exposed to EIHS, providing additional evidence of EIHS‐mediated impairment in kidney function (Goto et al., 2023).

We also discovered that EIHS changed several histological parameters linked to impaired kidney health, including vacuolization (Goto et al., 2022; Khan & Alden, 2002; Nonoyama & Fukuda, 2008). Objective determination of vacuolar cargo was beyond the scope of this investigation, but their appearance and the increased abundance of perilipin 2 and PPARγ raise the likelihood of lipid infiltration. The degree to which vacuoles might persist and how vacuole cargo might change owing to EIHS, and how those factors might contribute to acute and chronic renal injury, remains unknown. Nevertheless, although the changes induced herein resulted from an acute insult, it is notable that increased renal vacuolation has been reported in people with CKD (Nakamura et al., 2023), including those diagnosed with Mesoamerican nephropathy (Wijkström et al., 2013), a CKD not associated with traditional risk factors (e.g., diabetes, hypertension), which might be caused by repeated EIHS exposures (Chapman et al., 2021). We also discovered EIHS‐mediated alterations in renal corpuscle structure, such that the relative size of the glomerulus was increased and Bowman's space decreased. Glomerular enlargement has been associated with outcomes of kidney disease in both experimental models (Cahill et al., 1996; Fries et al., 1989; Miller et al., 1991) and humans (Fogo et al., 1990; Kataoka et al., 2011, 2012). Moreover, glomerular enlargement is common in Mesoamerican nephropathy, which could be secondary to widespread glomerulosclerosis, functional nephron loss and/or ischaemia (Wijkström et al., 2013), although such changes are driven by genuine remodelling with chronic disease, which is unlikely following an acute injury.

Given the relatively brief experimental period, it is unclear whether these changes represent transient adaptations or lasting remodelling akin to disease, although, given the totality of the data presented herein, including physical and biochemical changes, it seems likely that these changes are, at least in part, in response to an acute injury with an unknown resolution time. Ultimately, we interpret these changes as consequences of the acute effects of EIHS, although we are mindful of potential long‐term associations of thermic injury and CKD (Wang et al., 2019). Histological changes were accompanied by increased markers of tubular injury and inflammatory signalling. The activation of inflammatory signalling is likely to be a response to cellular injury (Zhang & Parikh, 2019), which has been reported owing to thermic injury of the kidney (Fischer et al., 2018; Hansson et al., 2020), and/or endotoxaemia, which is common during EIHS (Pearce et al., 2013a, 2013b). Our discovery of increased expression of TLR4, although puzzling, is likely to serve as an activator of inflammatory signalling. Consistent with these observations, others have reported that markers of kidney tubular injury are elevated during thermic injuries in humans without heatstroke (Chapman et al., 2020; Schlader et al., 2017), with heatstroke (Lin & Zhang, 2019; Schlader et al., 2022), and in patients with AKI and/or CKD (Zhang & Parikh, 2019). Collectively, the data presented herein, in addition to this previous work, provide compelling evidence that thermic injury caused by EIHS is sufficient to antagonize kidney health.

The underlying mechanisms contributing to renal water retention are not entirely clear. During EIHS, the kidney is simultaneously experiencing injury, reductions in blood flow to support thermoregulation and fluid conservation to promote the maintenance of body water in the presence of increased evaporative heat loss. It seems likely that the observed water retention is driven, at least in part, by thermic injury to the kidney and subsequent inflammation, which is supported by EIHS‐mediated inflammatory signalling (Bobkova et al., 2016; Sato et al., 2019; Wang et al., 2024). Alternatively, given EIHS‐mediated renal injury, there might be dysfunction and dysregulation of key sodium pumps/channels (Besse‐Eschmann et al., 2002; De Seigneux et al., 2006; Vogt & Favre, 1991) that cumulatively lead to exaggerated sodium retention and cause water retention in a manner similar to that described in the ‘overfill hypothesis’ (Bobkova et al., 2016). However, the role of overfill in EIHS renal water retention seems dubious, because it might require euhydration, which is uncertain even with ad libitum access to water. Determining the underlying cause of water retention is essential for the development of appropriate interventions to safeguard kidney health in the presence of EIHS.

In addition to injury, or perhaps owing to it, we also identified changes that might counter cell stress. Heat shock factor was increased by EIHS along with several HSPs, which we anticipate supports tolerance or resistance to thermal injury in the kidney (Emami et al., 1991; Sreedharan & Van Why, 2016). Of interest, we previously reported varied HSP responses to hyperthermia across a variety of tissues (Pearce et al., 2013a; Roths et al., 2023c; Yan et al., 2009), with a notably weak response in skeletal muscle, which might leave this tissue particularly sensitive to heat‐related damage. In our previous work, we discovered that EIHS impaired the degradation of autophagosomes in both skeletal muscle and cardiac muscle (Brownstein et al., 2017; Roths et al., 2023c). In tissues considered herein, several upstream autophagy regulatory proteins were increased, and at least one metric of flux was also increased, although we note that flux measured via accumulation of LC3 A/B II was similar between groups. Also, the canonical autophagy activator, p‐AMPK, was decreased by EIHS, despite the reduction in food intake and despite the potential for increased autophagy, raising the possibility of a non‐canonical activation of autophagy in addition to dysregulation of energy metabolism. Given these possibilities and our previous findings in muscle of impaired mitophagy, we further considered the extent to which EIHS altered markers of mitophagy. Consistent with a stress response, we found that the mitophagy markers BNIP and Parkin were increased by EIHS. Changes in fission and fusion proteins point to deliberate alterations in mitochondrial architecture; however, whether they support (fission) or oppose (fusion) mitophagy is difficult to ascertain. Finally, there was robust activation of ER stress in the kidneys from EIHS mice compared with TN mice. The accumulation of unfolded proteins can trigger the UPR and ultimately preserve or restore cell health and survival (Ricciardi & Gnudi, 2020). However, chronic ER stress and activation of the UPR actively participate in the progression of several pathological states, including the development of AKI (Morgan & Nicolas, 2018) and even muscle injury caused by dystrophin deficiency (Krishna et al., 2023). Furthermore, these changes are notably similar to EIHS‐mediated changes discovered in the heart (Roths et al., 2024). Nevertheless, the balance of autophagy and UPR in the progression of thermic injury or resistance to thermic injury is unknown and will be the focus of future investigation.

There are several limitations to this work that should be considered. First, only female mice were used in this investigation. We discovered in our pilot testing that females were more resistant to identical thermic stresses than males, which, although consistent with previous work using heat stroke models (Renteria et al., 2023), probably requires different heating conditions to elicit a similar degree of stress. Indeed, there is an apparent effect of biological sex on heat stress‐mediated outcomes in our porcine model using castrated males and age‐matched females (Rudolph et al., 2024a, 2024b, 2024c). Although beyond the scope of this investigation, biological sex will be considered in future work. Second, we did not control the oestrous cycle in this investigation; hence, we expect an array of phases to be represented in the dataset. Although this might obscure some outcomes, such randomness provides greater confidence in the changes detected herein. In future work, we will consider separately the impact of the oestrous and diestrous phases, because the differing hormonal milieu might impact the consequences of EIHS. Finally, circulating measures, such as BUN, are reported as a concentration and therefore could be impacted by apparent changes in blood volume should EIHS cause dehydration. Hence, these data should be interpreted with caution, and we advocate for future assessment of kidney function. Nevertheless, ignoring BUN as an indicator of kidney function, the sum total of the data reported herein indicates that EIHS impairs kidney health.

## 5 CONCLUSION

In summary, the intensity, duration and frequency of environmental heat and subsequent thermic injury are present and expanding human health challenges. The findings presented herein make it clear that EIHS injures the kidney and impairs kidney function. We also identified biochemical outcomes consistent with an EIHS‐mediated stress response; however, these results should be interpreted cautiously because they provide a snapshot of a highly dynamic system and are specific to the experimental conditions used in this study. As such, it is difficult to determine whether these changes support stress resistance or contribute to pathological outcomes. Undoubtedly, the data presented herein make it clear that EIHS impairs kidney health; however, further investigation is necessary to determine the extent to which EIHS‐mediated changes lead to lasting damage and impaired function, particularly within the context of underlying kidney disease.

## AUTHOR CONTRIBUTIONS

Conceptualization: Melissa Roths and Joshua T. Selsby. Formal analysis and interpretation: Melissa Roths, Alyona Michael, Zachary J. Schlader and Joshua T. Selsby. Funding acquisition: Joshua T. Selsby. Visualization: Melissa Roths and Joshua T. Selsby. Writing—original draft: Melissa Roths and Joshua T. Selsby. Writing—review and editing: Melissa Roths, Alyona Michael, Zachary J. Schlader and Joshua T. Selsby. All authors approved the final version of the manuscript and agree to be accountable for all aspects of the work in ensuring that questions related to the accuracy or integrity of any part of the work are appropriately investigated and resolved. All persons who are listed qualify for authorship, and all those who qualify for authorship are listed.

## CONFLICT OF INTEREST

The authors do not have conflicts to declare.

## Data Availability

All data are contained in the paper. Additional data from this investigation have been cited. For thoroughness, we anticipate the publication of an additional paper focused on skeletal muscle.
